# Higher detectability of gastric cancer after *Helicobacter pylori* eradication in texture and color enhancement imaging mode 2 in screening endoscopy

**DOI:** 10.1002/deo2.279

**Published:** 2023-07-30

**Authors:** Yuichiro Kemmoto, Shun‐ichiro Ozawa, Ryota Sueki, Keiichi Furuya, Daimon Shirose, Satoshi Wakao, Kuniaki Shindo, Atsushi Nagata, Tadashi Sato

**Affiliations:** ^1^ Department of Gastroenterology and Hepatology Japan Community Health Care Organization Yamanashi Hospital Yamanashi Japan; ^2^ Health Management Center Japan Community Health Care Organization Yamanashi Hospital Health Screening Center Yamanashi Japan

**Keywords:** endoscopy, gastric cancer, *Helicobacter pylori*, image‐enhanced endoscopy, texture and color enhancement imaging

## Abstract

**Objectives:**

The utility of texture and color enhancement imaging (TXI) in detecting gastric cancer (GC) has been investigated. However, few reports exist on TXI mode2 (TXI2) used for detecting GC; this study investigated the efficacy of TXI2 in GC detection during screening endoscopy.

**Methods:**

This study enrolled 13,440 participants with confirmed *Helicobacter pylori* (*H. pylori*) infection status who underwent screening endoscopy by 20 endoscopists in our health screening center. The participants were divided into two groups: one group was observed using white light imaging (WLI) only by 17 endoscopists (WLI group, 10,745 participants), and the other group was observed using TXI2 only by the other three endoscopists (TXI2 group, 2695 participants). We analyzed the detection rate and the characteristics of GC. In addition, considering the bias due to the diagnostic ability, we analyzed the subset of the WLI group where the participants were evaluated by the top three endoscopists based on their GC detection rate (Expert‐WLI group, 2792 participants) for comparison with the TXI2 group.

**Results:**

Fifty patients were diagnosed with GC. The GC detection rates were 0.68% and 0.71% in the Expert‐WLI and TXI2 groups, respectively. In patients who underwent screening endoscopy after *H. pylori* eradication, the detection rates of differentiated GC, L‐region lesions, and surface depressed‐type lesions were 0.52%, 0%, and 0.43% in the Expert‐WLI group and 1.36%, 0.78%, and 1.36% in the TXI2 group, respectively.

**Conclusions:**

In screening endoscopy, the detectability of differentiated GC and L‐region lesions and surface depressed‐type lesions after *H. pylori* eradication was higher in TXI2.

## INTRODUCTION

The *Helicobacter pylori* (*H. pylori*)‐negative population has become the majority in Japan because the incidence of *H. pylori* infection is declining and the eradication therapy for *H. pylori*‐infected gastritis has become widespread. The 5‐year survival rate of patients with advanced gastric cancer (GC) remains low^1^; however, the 5‐year survival rate in patients with early GC is better than 95%. Diagnosing GC in the early stage is important. The “Guidelines for Endoscopic Diagnosis of Early‐Stage Gastric Cancer (2019 Edition)” state the efficacy of image‐enhancement endoscopy for diagnosing GC in gastric endoscopic screening.^2^ However, the “Manual of Gastric Endoscopic Examination for Countermeasure Type Examination (2015 Edition)” states that image‐enhancement endoscopy is not necessary for diagnosing GCs in gastric endoscopic screening.^3^ A new image‐enhanced endoscopy, texture and color enhancement imaging (TXI), was developed based on the concept of emphasizing the contrast between normal gastric mucosa and lesions to improve the detectability of GC. TXI is an image‐enhancement method where the image obtained from white light imaging (WLI) is divided into a base image and a texture image; then, texture enhancement is applied to the texture image and brightness is applied to the base image. Reports have shown the usefulness of TXI in detecting atrophic mucosa and intestinal metaplasia and^4^, ^5^ gastric neoplasms^6^ and in improving the visibility of GC after *H. pylori* eradication.^7^ Particularly, in TXI mode 2 (TXI2), applying conventional diagnostics is easier, and TXI2 has been considered ideal for use in the first screening endoscopy.^8^ However, large‐scale studies showing the usefulness of TXI2 in detecting GC in screening endoscopy have not been conducted. Therefore, this study was designed to evaluate whether TXI2 improves the detectability of GC compared with WLI.

## MATERIALS AND METHODS

### Instruments

A newly developed video‐endoscopy system (EVIS X1; Olympus) and the ultrafine endoscope (GIF‐1200N, Olympus) were used. The structural enhancement function was set to A5 in the WLI and TXI2 modes.

### Study design and patients

**FIGURE 1:**
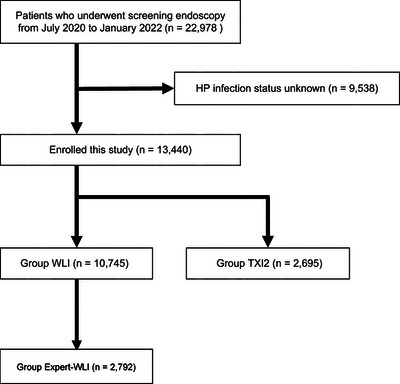
Flowchart of patient assignment

After excluding 9538 participants whose *H. pylori* infection status was uncertain, 13,440 participants who underwent endoscopic screening between July 2020 and January 2022 at our center were enrolled in this study. The group that was observed using WLI only in GC screening was designated the WLI group, whereas the group that was observed using only TXI2 was designated the TXI2 group (Figure 1). Twenty endoscopists participated in this study, of whom 17 were assigned to the WLI group and three to the TXI2 group. The number of endoscopists certified by the Japanese Society of Gastrointestinal Endoscopy in the WLI and TXI2 groups was 10 and 2, respectively. We retrospectively obtained participant information using questionnaires (i.e., age, sex, *H. pylori* antibody test, *H. pylori* eradication history, and *H. pylori* eradication results) and the medical records. We compared patient characteristics, *H. pylori*‐infection status, GC detection rate, biopsy success rate, time from *H. pylori* eradication to GC diagnosis, and endoscopic and histopathological findings (i.e., maximum diameter, localization, macroscopic findings, histopathological type, and depth of tumor invasion) between the WLI and TXI2 groups. The primary endpoint was the detection rate of GC using TXI2. The secondary endpoint was the characteristics of GC detected using TXI2. Furthermore, we established the Expert‐WLI group as a subset of the WLI group, comprising participants evaluated by the top three endoscopists who demonstrated the highest GC detection rate. We then proceeded to compare this group with the TXI2 group. Importantly, all three top‐performing endoscopists in the WLI group, based on their GC detection rate, held certification from the Japanese Society of Gastrointestinal Endoscopy (Figure 1). The Japan Community Healthcare Organization Yamanashi Hospital examined and registered this study (approval number 2020–4). All procedures were performed according to applicable rules and regulations. This was a single‐center observational study, and the “opt‐out” method was used because of the laws that protect personal information.

### Definition of *H. pylori* infection status and GC

*H. pylori* infection was diagnosed using serum IgG antibody (E‐Plate “Eiken” *H. pylori* antibody, Eiken Chemical, Tokyo, Japan), and cases with *H. pylori* antibody test results of 10 U/ml or higher were considered *H. pylori*‐positive,^9^, ^10^, ^11^ whereas those with *H. pylori* antibody test results of less than 10 U/ml were considered *H. pylori*‐negative. Cases of *H. pylori* infection were defined as *H. pylori*‐positive cases with no history of *H. pylori* eradication or those with unsuccessful *H. pylori* eradication. Among *H. pylori*‐negative cases, those with a history of *H. pylori* eradication were classified as post‐*H. pylori* eradication, whereas those without a history of *H. pylori* eradication were classified as *H. pylori*‐uninfected. GC diagnosed less than 1 year after *H. pylori* eradication was defined as GC with *H. pylori* infection, whereas GC detected more than 1 year after *H. pylori* eradication was defined as GC after *H. pylori* eradication.^12^ The duration between *H. pylori* eradication and GC diagnosis was defined as the time between the patient's negative urea breath test and stool *H. pylori* antigen test following *H. pylori* eradication treatment and the diagnosis of GC. After *H. pylori* eradication, patients with an *H. pylori* antibody test of less than 10 U/ml and no history of *H. pylori* eradication were considered spontaneously eradicated and treated for GC.

### Biopsy and pathological evaluation

Pathological diagnoses were made on biopsied tissue or resected specimens obtained during endoscopic resection or surgical removal. When both biopsy and resection specimens were available, the latter was used for the final diagnosis. Based on the group categorization, Group 4 (lesions classified as tumors but suspected to be cancer) and Group 5 (cancer) were identified as GC; however, Group 1–3 lesions were diagnosed as noncancers.

### Statistical analysis

Clinical data are expressed as percentages, means, medians, and ranges. The Kolmogorov–Smirnov test was used to test the normality of data distribution. Differences in proportions between the two groups were evaluated using the Mann–Whitney U test and Fisher's exact test. Continuous data were compared using the t‐test. All *p*‐values were two‐sided, with an alpha of 5% as the significance level. Continuous variables are expressed as medians or means and standard deviations. Taking into account the potential bias arising from the differences in diagnostic ability between the WLI group and the TXI2 group, we conducted an additional subgroup analysis comparing the Expert‐WLI and TXI2 groups. All statistical analyses were performed using EZR (Saitama Medical Center, Jichi Medical University), which is a graphical user interface for R (The R Foundation for Statistical Computing).

## RESULTS

### Patient's characteristics

The median participant age was 57 years (range, 24–93 years), and 8146 participants were male. The WLI group comprised 10,745 of the 13,440 patients, whereas the TXI2 group comprised 2695 patients. The median age was 57 years (range, 24–93 years) in the WLI group and 57 years (range, 28–92 years) in the TXI2 group. Regarding the infection status, 399 and 6123 patients in the WLI group and 108 and 1556 patients in the TXI2 group were infected and uninfected with *H. pylori*, respectively, whereas *H. pylori* was eradicated in 4223 patients in the WLI group and 1031 patients in the TXI2 group, respectively. No significant difference in the patient background was observed between the two groups (Table 1).

**TABLE 1:** Clinical characteristics of the participants who underwent screening endoscopy.

	WLI group, *n* = 10,745	TXI2 group, *n* = 2695	Total, *n* = 13440	*p*‐value
Age, years, median (range)	57 (24–93)	57 (28–92)	57 (24–93)	0.43
Sex (%)				
Male	6495 (60.4)	1651 (61.3)	8146 (60.6)	0.44
Female	4250 (39.6)	1044 (38.7)	5294 (39.4)	0.44
*H. pylori* status (%)				
Eradicated	4223 (39.3)	1031 (38.3)	5254 (39.1)	0.33
Infected	399 (3.7)	108 (4.0)	507 (3.8)	0.46
Uninfected	6123 (57.0)	1556 (57.3)	7679 (57.1)	0.49
Smoking (current or past smoker) (%)	5249 (48.9)	1341 (49.8)	6590 (49.0)	0.40
Drinking (7 units/week more) (%)	2929 (27.3)	748 (27.8)	3677 (27.4)	0.61

Abbreviations: *H. pylori*, *Helicobacter pylori*; TXI2, texture and color enhancement imaging mode2; WLI, white light imaging.

### GC detection and biopsy rates

In this study, 50 (0.37%) GCs were diagnosed (49 early‐stage and one advanced). The detection rate of GC was 0.29% (31/10.745) in the WLI group and 0.71% (19/2,695) in the TXI2 group, showing a significant difference (*p* = 0.004; odds ratio [OR] = 2.45). The detection rate of Group 3 lesions was 0.1% (11/10,745) in the WLI group and 0.18% (5/2695) in the TXI2 group, with no significant difference (*p* = 0.34). No significant difference was observed in the rate of biopsy cases (*p* = 0.26); however, the positive predictive value of biopsy was 4.9% and 11.0% in the WLI and TXI2 groups, respectively (*p* = 0.007; OR = 2.37; Table 2).

**TABLE 2:** Gastric cancer detection rate, biopsy rates, and duration from *Helicobacter pylori* (*H. pylori*) eradication to GC diagnosis

	WLI group, *n* = 10,745	TXI2 group, *n* = 2695	Total, *n* = 13,440	*p*‐value
Biopsy				
Cases of biopsy, *n*, (%)	628 (5.8)	173 (6.4)	801 (6.0)	0.26
Positive predictive value of biopsy, %, (*n*)	4.9 (31/628)	11.0 (19/173)	6.2 (50/801)	0.007
Diagnosed GCs (%)	31 (0.29)	19 (0.71)	50 (0.37)	0.004
Diagnosed Group3 lesions (%)	11 (0.10)	5 (0.18)	16 (0.12)	0.34
*H. pylori* status of GCs, %, (*n*)				
Eradicated	0.43 (18/4223)	1.36 (14/1031)	0.61 (32/5254)	0.002
Infected	2.0 (8/339)	0.93 (1/108)	2.0 (9/447)	0.69
Uninfected	0.08 (5/6123)	0.26 (4/1556)	0.11 (9/7679)	0.08
Duration between eradication and diagnosis, month, mean (±SD)	52.3 (±21.4)	80.9 (±16.9)	64.0 (±23.5)	0.001

Abbreviations: GC, gastric cancer; *H. pylori*, *Helicobacter pylori*; TXI2, texture and color enhancement imaging mode2; WLI, white light imaging.

### GC detection rate after *H. pylori* eradication and the duration from *H. pylori* eradication to GC diagnosis

The detection rate of GC after *H. pylori* eradication was 0.43% (18/4223) in the WLI group and 1.36% (14/1031) in the TXI2 group, showing a significant difference (*p* = 0.002; OR = 3.21). The duration from *H. pylori* eradication to GC diagnosis was 64 ± 23.58 months. The duration was 52.3 ± 21.4 months in the WLI group and 80.9 ± 16.9 months in the TXI2 group, showing a significant difference (*p* = 0.001; Table 2).

### Endoscopic and histopathological findings

Comparing the detection rate of GC according to lesion location, the detection rate in the L‐region was 0.11% (12/10,745) in the WLI group and 0.45% (12/2695) in the TXI2 group, showing a significant difference (*p* < 0.001; OR = 3.99). Comparing the detection rate of GC according to macroscopic classification, the detection rate for surface depressed‐type (0–IIc) lesions was 0.19% (20/10,745) in the WLI group and 0.63% (17/2695) in the TXI2 group, showing a significant difference (*p* < 0.001; OR = 3.40). For GCs after *H. pylori* eradication, the detection rates for the L‐region and 0–IIc lesions were also significantly higher in the TXI2 group (Table 3). No significant difference in the tumor size of the diagnosed GC was observed (*p* = 0.09). However, the detection rate of GC <10 mm was 0.11% (12/10,745) in the WLI group and 0.45% (12/2,695) in the TXI2 group, showing a significant difference (*p* < 0.001; OR = 3.21). Comparing the detection rate of GC according to histological type, the detection rate of the differentiated GC was 0.59% (16/2695) in the WLI group and 0.22% (24/10,745) in the TXI2 group, showing a significant difference (*p* = 0.004; OR = 2.66). Comparing the GC detection rate according to the depth of invasion, no significant difference in the detection rate of GC deeper than pT1b (*p* = 1). However, the detection rate of GC in pT1a was 0.22% (24/10,745) in the WLI group and 0.67% (18/2695) in the TXI2 group, showing a significant difference (*p* < 0.001; OR = 3.00; Table 4).

**TABLE 3:** Characteristics of the diagnosed gastric cancer.

Total GC cases	WLI group, *n* = 10,745	TXI2 group, *n* = 2695	Total, *n* = 13,440	*p*‐value
Location (%)				
U	5 (0.05)	3 (0.11)	8 (0.06)	0.20
M	14 (0.13)	4 (0.15)	18 (0.13)	0.77
L	12 (0.11)	12 (0.45)	24 (0.18)	<0.001
Paris classification (%)				
0–I	0	0	0	NA
0–IIa	4 (0.04)	1 (0.04)	5 (0.04)	1.00
0–IIb	6 (0.06)	1 (0.04)	7 (0.05)	1.00
0–IIc	20 (0.19)	17 (0.63)	37 (0.28)	<0.001
0–III	1 (0.01)	0	1 (0.007)	1.00

GC cases after *H. pylori* eradication	WLI group, *n* = 4223	TXI2 group, *n* = 1031	Total, *n* = 5254	
Location (%)				
U	2 (0.05)	2 (0.19)	4 (0.08)	0.17
M	10 (0.24)	3 (0.29)	13 (0.25)	0.73
L	6 (0.14)	9 (0.87)	15 (0.29)	<0.001
Paris classification (%)				
0–I	0	0	0	NA
0–IIa	2 (0.05)	0	2 (0.04)	1.00
0–IIb	2 (0.05)	0	2 (0.04)	1.00
0–IIc	13 (0.30)	14 (1.36)	27 (0.51)	<0.001
0–III	1 (0.02)	0	1 (0.02)	1.00

Location (U, cardia, fornix and upper body; M, middle body and lower body; L, anglus, antrum, and pylorus)

Abbreviations: GC, gastric cancer; *H. pylori*, *Helicobacter pylori*; NA, not applicable; TXI2, texture and color enhancement imaging mode2; WLI, white light imaging.

**TABLE 4:** Pathological data.

	WLI group, *n* = 10,745	TXI2 group, *n* = 2695	Total, *n* = 13,440	*p*‐value
Maximum tumor diameter, mm, mean (±SD)	12.4 (±9.4)	8.5 (±4.37)	10.93 (±8.01)	0.09
Diagnosed GC <10 mm (%)	12 (0.11)	12 (0.45)	24 (0.19)	**<0.001**
Histological findings (%)				
Differentiated: tub1/tub2	20 (0.22)	15 (0.59)	35 (0.26)	**0.002**
Differentiated: GA‐FG	4 (0.04)	1 (0.04)	5 (0.04)	1.00
Undifferentiated: por/sig	7 (0.07)	3 (0.11)	10 (0.07)	0.43
Depth of invasion (%)				
pT1a	24 (0.22)	18 (0.67)	42 (0.31)	**<0.001**
pT1b and below	7 (0.07)	1 (0.04)	8 (0.06)	1.00

Histological findings (tub1, well‐differentiated; tub2, moderately differentiated adenocarcinoma; GA‐FG, gastric adenocarcinoma of fundic gland type; por, poorly differentiated adenocarcinoma; sig, signet ring cell carcinoma).

Abbreviations: GC, gastric cancer; NA, not applicable; TXI2, texture and color enhancement imaging mode2; WLI, white light imaging.

### Comparison between the Expert‐WLI and TXI2 groups

Next, we performed additional analyses of characteristics that were significantly different between the TXI2 and WLI groups (GC detection rate, tumor size, macroscopic classification, lesion location, and rate of differentiated GC) by comparing the TXI2 group with the Expert‐WLI group. The Expert‐WLI group included 2792 patients, among whom *H. pylori* was eradicated in 1152 patients. The detection rate of GC was 0.68% (19/2792) in the Expert‐WLI group and 0.71% (19/2695) in the TXI2 group, with no significant difference (*p* = 1). In addition, the detection rate of GC was not significantly different among patients after *H. pylori* eradication (*p* = 0.21). However, in an analysis limited to differentiated GC (tub1, tub2) after *H. pylori* eradication, the detection rate of GC was 0.52% (6/1152) in the Expert‐WLI group and 1.36% (14/1031) in the TXI2 group, with a significant difference (*p* = 0.045; OR = 2.63). The detection rate of GC <10 mm was 0.17% (2/1152) in the Expert‐WLI group and 0.69% (7/1031) in the TXI2 group, which tended to be higher in the TXI2 group (*p* = 0.09). Similarly, the detection rate of pT1a was 0.43% (5/1152) in the Expert‐WLI group and 1.26% (13/1031) in the TXI2 group, which tended to be higher in the TXI2 group (p = 0.055). The detection rate of L‐region lesions was 0 in the Expert‐WLI group and 0.78% (8/1031) in the TXI2 group, with a significant difference (*p* = 0.002). Finally, the detection rate of 0–IIc lesions was 0.43% (5/1152) in the Expert‐WLI group and 1.36% (14/1031) in the TXI2 group, with a significant difference (*p* = 0.022; OR = 3.16; Table 5).

**TABLE 5:** Expert‐white light imaging (WLI) group versus texture and color enhancement imaging mode2 group.

Total GC cases	Expert‐WLI group, *n* = 2792	TXI2 group, *n* = 2695	Total, *n* = 5487	*p*‐value
Diagnosed GCs (%)	19 (0.68)	19 (0.71)	38 (0.69)	1
Diagnosed GC <10 mm (%)	11 (0.39)	12 (0.45)	23 (0.42)	0.84
Depth of invasion, pT1a (%)	16 (0.57)	18 (0.67)	34 (0.62)	0.73
Location, L lesion (%)	7 (0.25)	12 (0.45)	19 (0.35)	0.25
Paris classification, 0–IIc (%)	11 (0.39)	17 (0.63)	28 (0.51)	0.26

GC cases after *H. pylori* eradication	Expert‐WLI group, *n* = 1152	TXI2 group, *n* = 1031	Total, *n* = 2183	
Diagnosed GCs (%)	9 (0.78)	14 (1.36)	23 (1.05)	0.21
Diagnosed GC <10 mm (%)	5 (0.43)	7 (0.69)	12 (0.55)	0.56
Depth of invasion, pT1a (%)	7 (0.61)	13 (1.26)	20 (0.92)	0.12
Location, L lesion (%)	2 (0.17)	9 (0.87)	11 (0.50)	**0.03**
Paris classification, 0–IIc (%)	5 (0.43)	14 (1.36)	19 (0.87)	**0.022**

Differentiated GC (tub1, tub2) cases after *H. pylori* eradication	Expert‐WLI group, *n* = 1152	TXI2 group, *n* = 1031	Total, *n* = 2183	
Diagnosed GCs (%)	6 (0.52)	14 (1.36)	20 (0.92)	**0.045**
Diagnosed GC <10 mm (%)	2 (0.17)	7 (0.69)	9 (0.41)	0.09
Depth of invasion, pT1a (%)	5 (0.43)	13 (1.26)	18 (0.82)	0.055
Location, L lesion (%)	0	8 (0.78)	8 (0.37)	**0.002**
Paris classification, 0–IIc (%)	5 (0.43)	14 (1.36)	19 (0.87)	**0.022**

Location (L, anglus, antrum, and pylorus).

Histological findings (tub1, well differentiated; tub2, moderately differentiated adenocarcinoma).

Abbreviations: GC, gastric cancer; *H. pylori*, *Helicobacter pylori*; TXI2, texture and color enhancement imaging mode2; WLI, white light imaging.

## DISCUSSION

**FIGURE 2:**
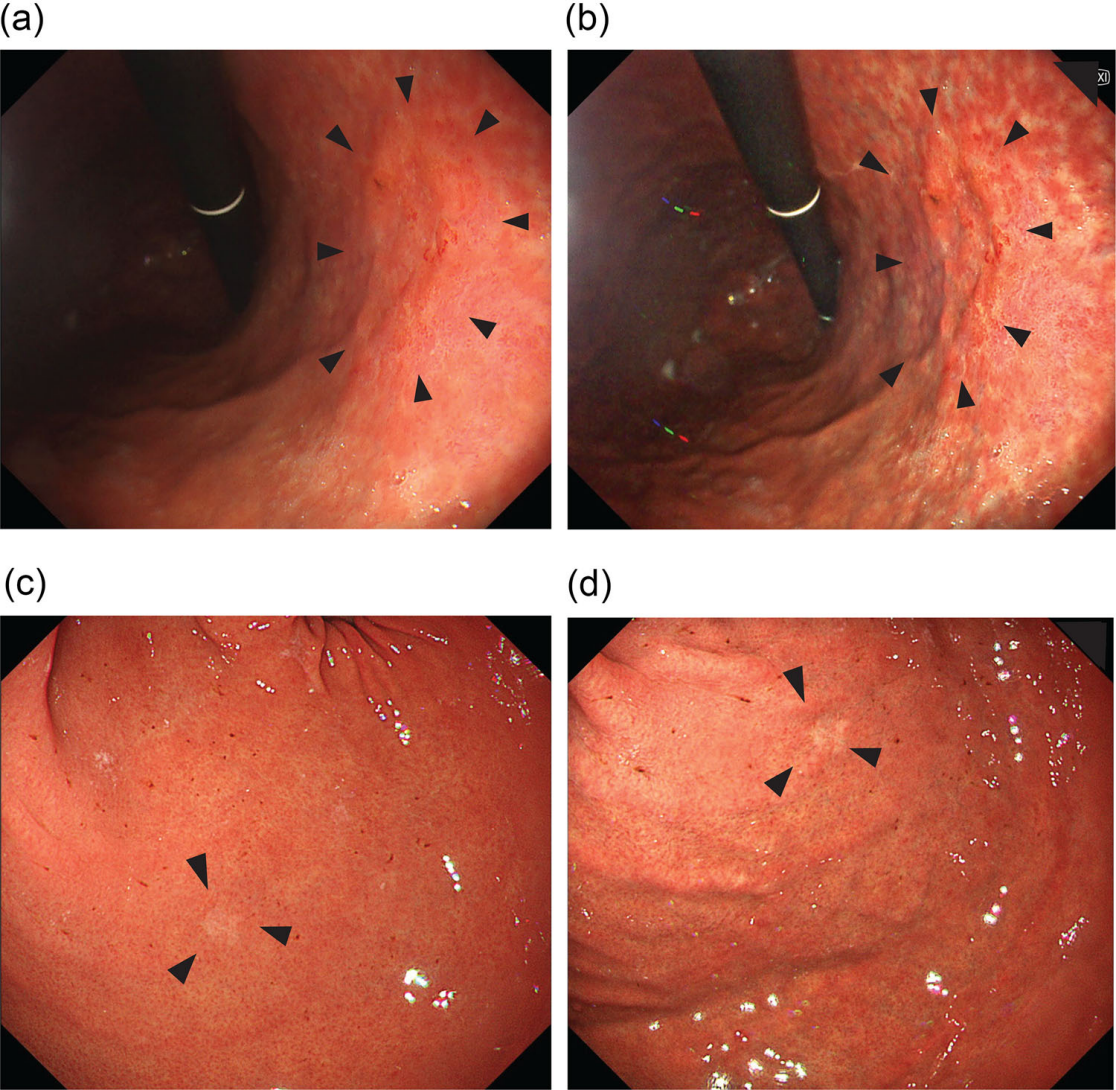
Case of early gastric cancer for which structure‐ and brightness‐enhanced texture and color enhancement imaging mode2 (TXI2) images create a distinct contrast against the surrounding mucosa. Case 1. Well‐differentiated tubular adenocarcinoma after *Helicobacter pylori* eradication. (a) White light imaging. (b) TXI2. The application of TXI2 accentuates mucosal irregularities, presenting lesions in three dimensions. Case 2. Signet ring cell carcinoma. (c) White light imaging . (d) TXI2. The contrast with the surrounding mucosa is distinct by enhancement of color and structure.

Because the *H. pylori*‐infection rate has recently declined and the eradication therapy has become widespread, the proportion of *H. pylori*‐eradicated and *H. pylori*‐uninfected subjects has increased in endoscopic screening, and endoscopic findings of GC have varied depending on the *H. pylori* infection status. Furthermore, the development of novel modalities, such as image‐enhanced endoscopy, is progressing, and new knowledge of endoscopic diagnosis is required for detecting GC. In linked color imaging (LCI) using the LASEREO system (Fujifilm, Tokyo), GC has high detectability compared with that in WLI.^13^, ^14^, ^15^, ^16^, ^17^, ^18^ A new image‐enhancement technique, TXI, is also expected to improve the detectability of lesions. Reports on TXI have shown improved visibility of gastric neoplasms^6^ and GC after *H. pylori* eradication.^7^ However, large‐scale studies showing the usefulness of TXI in detecting GC in screening endoscopy have not been conducted. In this study, TXI2 improved the detection rate of GC in endoscopic screening. Therefore, TXI2 is useful in GC screening. To the best of our knowledge, this is the first study to describe the clinical efficacy of TXI2 in GC screening. Endoscopists usually detect GC by detecting the border nature of the lesion with mucosal irregularities and color tone changes and by concentrating the contrast on the surrounding mucosa. A recent study reported that by analyzing objective color tone, TXI improved the visibility of gastric neoplasms, such as GC and gastric adenoma, more than WLI.^19^, ^20^ TXI1 has been reported to improve the visibility of lesions compared with WLI.^19^, ^20^ However, TXI2 is similar to WLI tone compared with TXI1 which is more color enhanced. Therefore, TXI2 makes it easier to apply existing diagnostics. In this study, we showed that screening with TXI2 improved the detection rate of gastric neoplasms by adding structural and brightness enhancements (Figure 2). In particular, our analysis also showed that TXI2 improved the detectability of differentiated GC (tub1, tub2) after *H. pylori* eradication as well as the detectability of L‐region and 0–IIc lesions.

**FIGURE 3:**
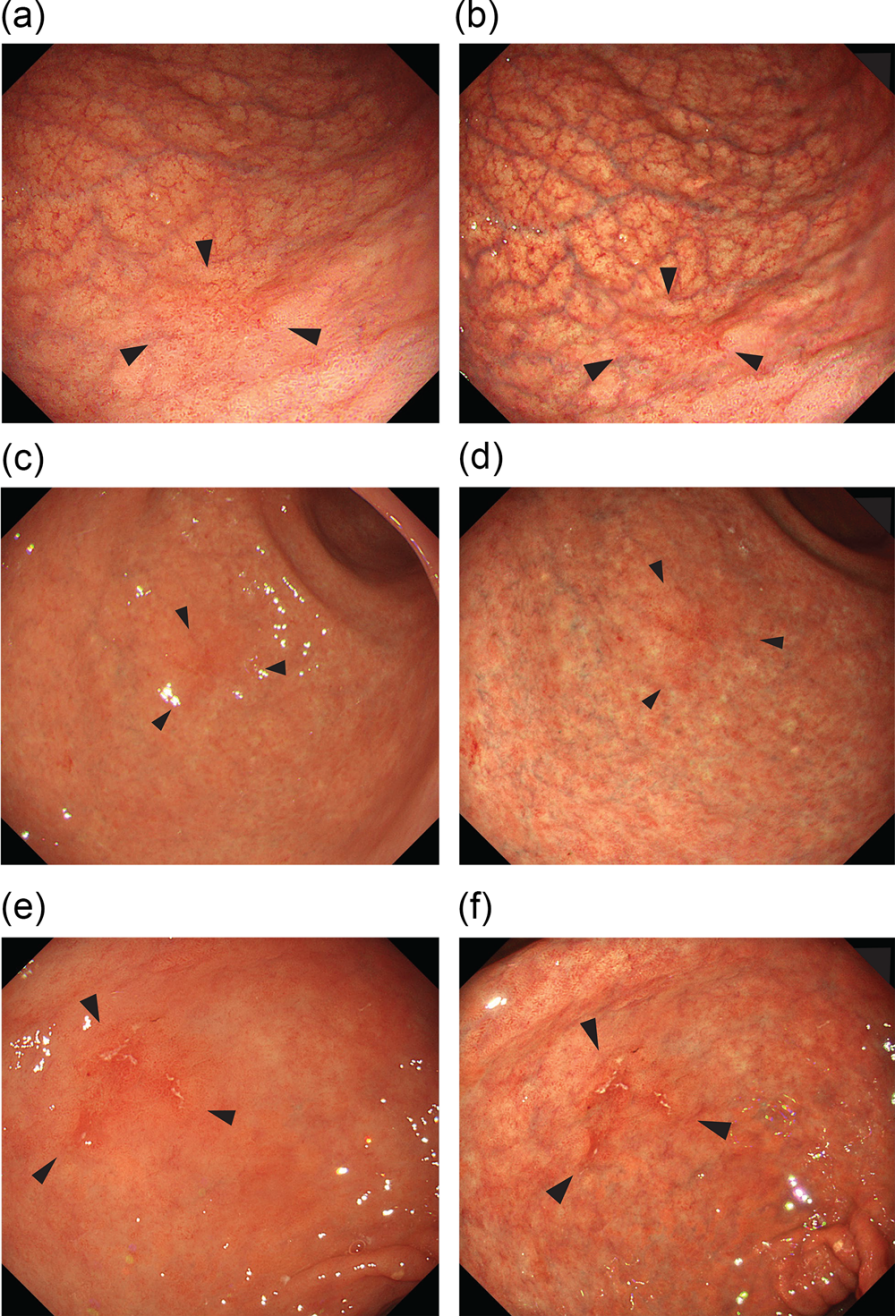
Cases of early gastric cancer, tub1 after *Helicobacter pylori* eradication in which texture and color enhancement imaging mode2 (TXI2) improved the atrophic mucosa around the tumor and enabled its detection. Case 1. Lower body, tub1 (a) white light imaging. (b) TXI2; Case 2. Lower body, tub1 (c) white light imaging. (d) TXI2; Case 3. Antrum, tub1 (e) white light imaging. (f) TXI2.

In the cases after *H. pylori* eradication, the recognition of lesions is considerably affected by background mucosal changes due to eradication. *H. pylori* eradication has been shown to improve inflammation of the stomach, which causes changes, such as enlarged folds, mucosal swelling, sticky mucous, and diffuse redness.^21^, ^22^ In contrast, *H. pylori* eradication obscures the border between the lesion and the surrounding mucosa by flattening the tumor due to the appearance of non‐neoplastic lesions and epithelium with low‐grade atypia.^23^ In this study, the detection rate of differentiated GC (tub1, tub2) after *H. pylori* eradication was higher in the TXI2 group, particularly in the L‐region and 0–IIc lesions. Additionally, the time from *H. pylori* eradication to GC diagnosis was significantly longer in the TXI2 group. It has been reported that the detection rate of atrophy and intestinal metaplasia was increased when using TXI^20^, ^21^ and that TXI improves the visibility of 0–IIc lesions.^19^ We suggested that the detection rate improved because of the contrast with atrophic mucosa around the lesion due to TXI2's ability to enhance color and structure (Figure 3).

This study was performed in a real‐world clinical GC screening setting, so there are several limitations. First, it was a single‐center study; second, it was a retrospective study, and the degree of mucosal atrophy after *H. pylori* eradication could not be assessed in all cases; third, the modality was wholly associated with each endoscopist. We did a subgroup analysis to overcome this issue. However, we could not eliminate the influence of the quality of endoscopists against the detection rate of GC in each modality; and fourth, the figures were based on the total number of patients who underwent endoscopic screening. However, screening using TXI2 has actually improved the detection rate of GC after *H. pylori* eradication and is expected to be useful. Future studies with a larger sample size and a prospective multicenter randomized controlled trial to assess the efficacy of TXI2 in the detectability of gastric neoplasms are required. Moreover, it is important to note that no cases of TXI mode1 (TXI1) were observed in this study, which hindered our ability to conduct a comparative analysis among TXI1, TXI2, and WLI. In future research, it is essential to assess and compare the effectiveness of both TXI2 and TXI1 in detecting gastric neoplasms.

In conclusion, this study showed that TXI2 was useful in GC screening for cases after *H. pylori* eradication because it allowed the detection of small variations between the surrounding mucosa and the actual lesion. TXI2 may be particularly useful for L‐region and 0–IIc lesions. In addition, comprehending the characteristics of these image‐enhancement methods is expected to improve GC diagnostic performance after *H. pylori* eradication.

## CONFLICT OF INTEREST STATEMENT

The authors declare no conflict of interest.
